# Wearables as a tool for measuring therapeutic adherence in behavioral health

**DOI:** 10.1038/s41746-021-00458-9

**Published:** 2021-05-10

**Authors:** Leia Wedlund, Joseph Kvedar

**Affiliations:** 1 Harvard Medical School, Boston, MA USA; 2 Mass General Brigham, Boston, MA USA

**Keywords:** Preventive medicine, Predictive markers

Medication nonadherence touches every patient population, driving increased healthcare costs^1^, and negatively affecting health outcomes^2^. The issue can be particularly severe amongst patients with mental illness. It is estimated that only 1 in 3 schizophrenia patients is fully adherent to their medication regimen, and nonadherence accounts for 40% of schizophrenia rehospitalization costs^3^.

When the FDA approved digital aripiprazole (an antipsychotic medication containing an ingestible sensor to track consumption), the medical community gained a new tool for measuring compliance, theoretically paving the way for new initiatives to find and help nonadherent patients. However, digital aripiprazole comes with a hefty price tag of $1700 for a 30-day supply (85× the price of generic aripiprazole)^4^, and with no existing randomized control trials to show how digital aripiprazole changes medication adherence^5^, it may be difficult to justify the cost.

Interestingly, Otsuka (the very pharmaceutical company behind digital aripiprazole) has funded a new study that suggests we may be able to estimate psychiatric medication adherence in a much less costly fashion. A recent paper by Cochran et al. describes how basic heart rate and accelerometer data (the kind collected by an average smart watch) can predict, which patients are most likely to adhere to their psychiatric medication regimen^6^.

In the study, 95 patients with schizophrenia, bipolar disorder, and major depressive disorder, all on stable doses of aripiprazole, were provided with a supply of digital aripiprazole for several weeks. Digital aripiprazole comes with a wearable patch that must be placed on the skin to detect, when the sensors in the pills are ingested. Cochran and colleagues used these same patches to collect heart rate and accelerometer data, giving them information about each participant’s activity levels throughout the study. The digital aripiprazole sensors provided an objective measure of medication adherence, which was used to determine how well heart rate and accelerometer data could predict adherence.

The accelerometers in each patch allowed investigators to calculate an “activity rhythm score” capturing how consistent a person’s step count was from day-to-day. They also calculated a “relative active-interval heart rate” which indicated the intensity of physical activity. Both a high activity rhythm score (indicating that a person’s step count was consistent with their routine), and a high relative active-interval heart rate (indicating intense physical activity as compared to baseline), were correlated with the likelihood that a study participant would take their medications the next day. When evaluating medication adherence (as measured by the digital aripiprazole sensors) over several weeks persons with a high activity rhythm score, who were consistent in their daily physical activity (i.e., step count) had significantly higher medication adherence than those with a low activity rhythm score^6^.

This novel work linking activity levels to medical adherence paves the way for new studies that seek to not only predict, but also increase, therapy utilization. Given the well-established link between exercise and mental health^7^, it is possible that consistent physical activity not only correlates with adherence, but also causally affects it in behavioral health patients. If further study supports this hypothesis, the type of basic wearables data collected in this study may one day provide both valuable predictive information and a foundation for personalized and specific therapeutic lifestyle changes.
